# A case report: Catatonic symptoms secondary to systemic lupus erythematosus with multiple infections: neuropsychiatric or “mimickers?”

**DOI:** 10.1097/MD.0000000000033746

**Published:** 2023-06-09

**Authors:** Lizeyu Lv, Yong Lin, Yu Zhang, Wen Xiao, Mingquan Li, Liangbin Zhao

**Affiliations:** a School of Clinical Medicine, Chengdu University of Traditional Chinese Medicine, Chengdu, China; b Department of Neuropsychiatrics, Hospital of Chengdu University of Traditional Chinese Medicine, Chengdu, China; c Department of Nephrology, Hospital of Chengdu University of Traditional Chinese Medicine, Chengdu, China.

**Keywords:** case report, catatonic, neuropsychiatric systemic lupus erythematosus, systemic lupus erythematosus

## Abstract

Systemic lupus erythematosus (SLE) is frequently accompanied by neuropsychiatric (NP) manifestations. However, typical symptoms of catatonia are uncommon. Neuropsychiatric SLE or its “mimickers” may cause NP symptoms, making differential diagnosis a significant challenge in clinical practice.

**Patient concerns:**

A 68-year-old female with SLE was hospitalized for edema, lung infection, and recurrent fungal mouth ulcers after multiple courses of cortisol and immunosuppressive therapy. Five days after admission, stupor, immobility, mutism, and rigidity were observed.

**Diagnosis:**

“Mimickers”: catatonic disorder due to a general medical condition.

**Intervention:**

Initially, relevant laboratory tests, imaging studies, and the disease activity index score were performed. A survey of the causes of the disease was conducted among the patient’s relatives. Subsequently, we discontinued moxifloxacin, corticosteroids, fluconazole, and other medications and inserted a gastric tube for nutritional support. During this process, traditional Chinese medicine and acupuncture have been utilized.

**Outcomes:**

After 3 days, the patient recovered and only complained of fatigue.

**Conclusion:**

When SLE presents with NP symptoms, it is essential to make a correct diagnosis in order to guide appropriate treatment by actively searching for inducers and clinical, laboratory, and neuroradiological characteristics that can aid in the differential diagnosis. When treatment options are limited, it can be beneficial to try a variety of combination strategies, such as traditional Chinese medicine and acupuncture.

## 1. Introduction

Systemic lupus erythematosus (SLE) is a chronic multisystem autoimmune disease with a wide range of clinical manifestations and a complicated pathogenesis, which frequently presents as neuropsychiatric (NP) manifestations.^[1]^ In this disease, however, typical catatonic symptoms such as stupor, rigidity, mutism, and immobility are uncommon. Clinically, NP symptoms may be caused by neuropsychiatric systemic lupus erythematosus (NPSLE) or its “mimickers” such as infections, drug-induced side effects, metabolic abnormalities, malignancies, alcohol-related disorders, etc. According to the heterogeneity of clinical manifestations, the estimated prevalence of NPSLE ranges from 37% to 95%.^[2]^ Indeed, it is the diversity and complexity of etiologies that make the differential diagnosis of the disease a major challenge in clinical practice.

## 2. Case presentation

**Figure 1.**
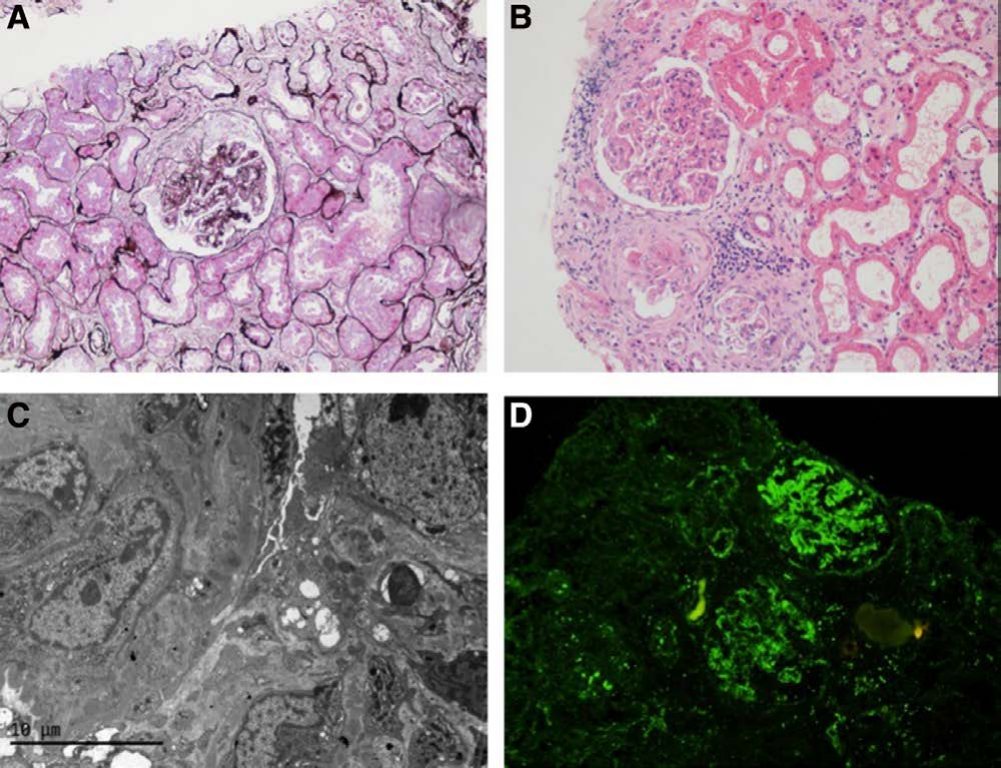
The pathological biopsy diagnosis of diffuse proliferative lupus nephritis with partial crescent formation and acute tubular injury by renal needle aspiration was made [type IV, ISN/RPS classification]; AI:1/24; CI:3/12; A: PASM staining: cellular fibrous crescent formation was seen, and some capillary lumens were narrow. B: HE staining: on light microscopy, fibrous crescent formation, moderate diffuse hyperplasia of small globular mesangial cells, and stroma with focal segmental severe hyperplasia. C: Electron microscope: basement membrane: segmental thickening; Podocyte: foot process segmental fusion. D: Immunofluorescence or histochemistry: C3++~+++.

A 68-year-old female with SLE was hospitalized with edema, cough, shortness of breath, and recurrent oral ulcers. Prior to this event, the suffering of generalized edema, abnormalities in kidney function, and massive proteinuria prompted her to undergo a renal biopsy, which revealed lupus nephritis (Fig. 1). Six intravenous treatments with methylprednisolone and cyclophosphamide were administered to the patient around 2021, followed by oral prednisone (35 mg/d) and hydroxychloroquine (0.4 g/d) for maintenance. During the history-taking process, except for hypertension, she denied any history of other chronic diseases, including coronary heart disease, diabetes, epilepsy, etc. The physical examination revealed both moist rales in the lungs and edema of the lower extremities. As for the supplementary examination, she had an elevated C-reactive protein level of 201.81 mg/dL, decreased hemoglobin (75 g/L), and albumin (34.2 g/L), and a fungal D-glucan test result of 147.65 pg/mL. On subsequent microbial cultures, oral secretions yielded Candida albicans, and sputum cultures yielded Pseudomonas aeruginosa. A chest computed tomography scan revealed an infection as well.

**Figure 2.**
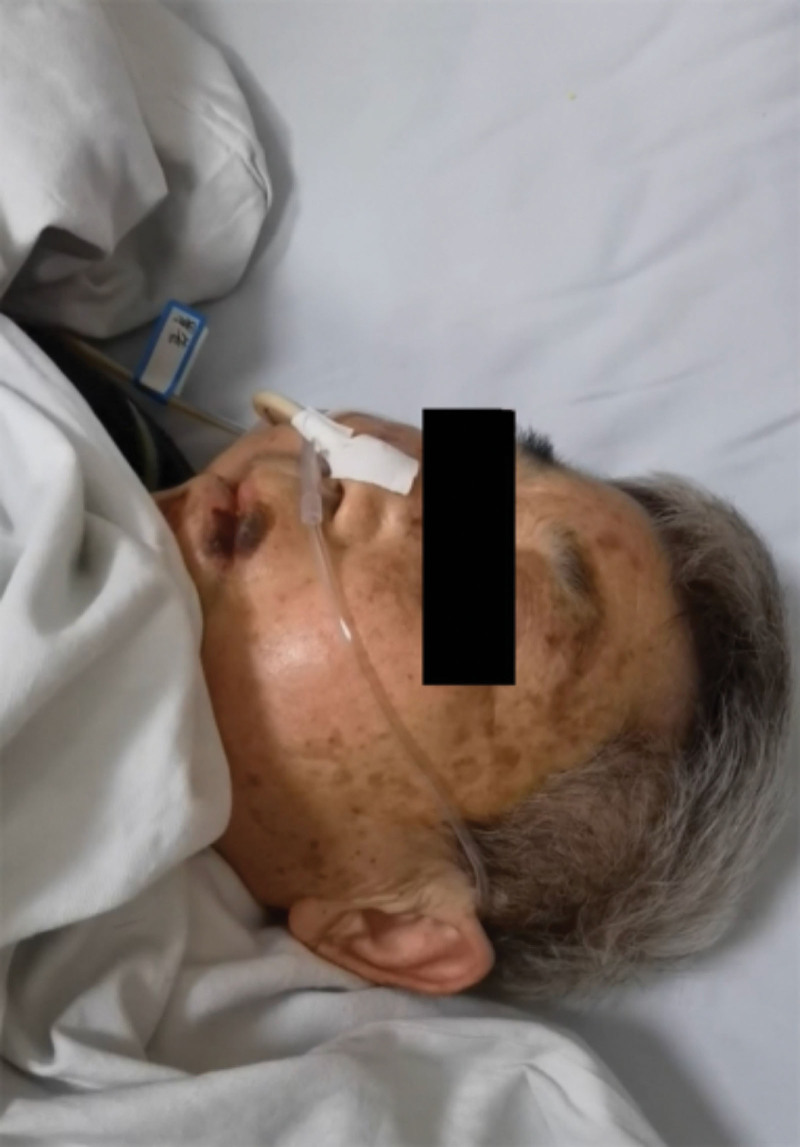
The state of the patient at the time of the disease: stupor, immobility, mutism, and rigidity.

On the fifth day of hospitalization, she was found to be in a state of stupor, immobility, mutism, and rigidity, just like the typical catatonic symptoms (Fig. 2). Her vital signs, however, were stable, and a physical examination revealed that she was sensitive to the light reflex, corneal reflex, painful stimuli, and a strong grip reflex. Signs of meningeal irritation and pathological signs were negative, and the muscle tone of all 4 limbs was not diminished.

**Figure 3.**
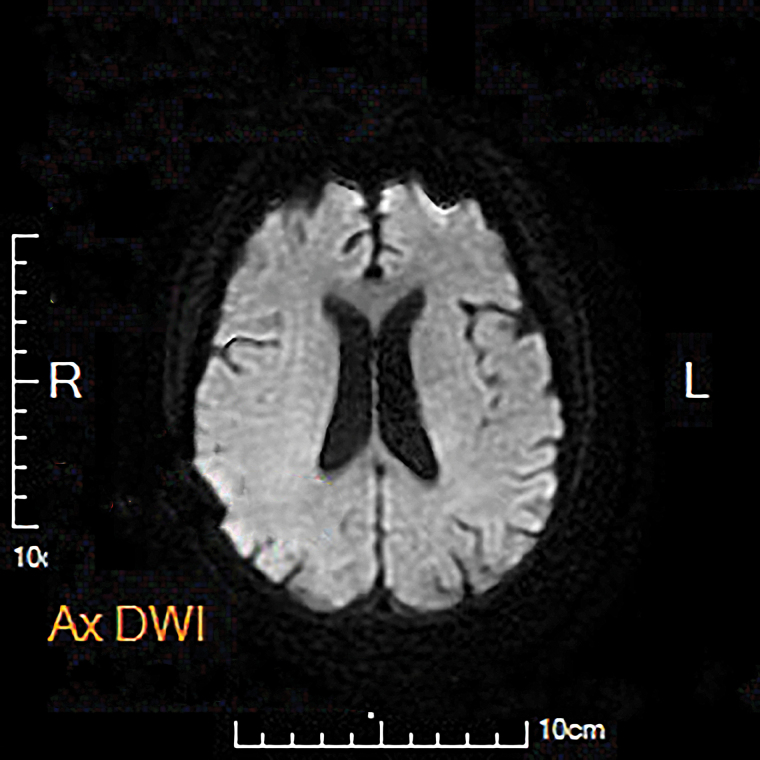
MRI diffusion-weighted image shows no acute changes. MRI = magnetic resonance imaging.

Given the complexity of her SLE, we initially suspected she had NPSLE. To further rule out potential causes of the symptoms, we immediately performed the following tests and examinations: electrolytes, liver function, renal function, procalcitonin, blood routine, blood gas analysis, cranial magnetic resonance imaging (MRI), electroencephalography, and cerebrospinal fluid (CSF) examination. Meanwhile, we detected autoantibodies, including anti-ribosomal-P (2), anti-phospholipase-A2-receptor, and anti-NMDA receptor antibodies, all within the normal range. We also resulted the SLE disease activity index (SLEDAI-2000) score for moderate levels and sought neurology advice. However, the relevant results and recommendations did not provide particularly clear diagnostic evidence for NPSLE (Tables 1–3; Fig. 3). We stopped all previous oral medications, including hydroxychloroquine, prednisone, and fluconazole, and the intravenously administered antibiotic was also changed from the suspected moxifloxacin to azithromycin. A gastric tube was inserted for provisional nutritional support. However, the patient’s clinical improvement was insignificant on the first day. Notably, we actively inquired about the situation and discovered that the patient had a violent quarrel with her family prior to the onset of the symptoms, and her mood fluctuated greatly, so we requested a consultation for psychiatric assessment, and a Bush-Francis Catatonia Rating Scale (BFCRS) score of 15 out of 62 was determined. After ruling out the majority of parenchymal diseases, we determined that catatonic disorder due to a general medical condition was more likely than NPSLE. Concerns regarding renal function impairment and the potential for further deterioration of the coma were discussed. We finally decided to temporarily hold off on benzodiazepines (BZDs), the first-line treatment for catatonia. Based on this, we started to apply traditional Chinese medicine (TCM) and acupuncture reasonably for adjuvant therapy (specific TCM and acupuncture prescriptions are shown in Tables 4 and 5). On the third day following the event, the patient recovered, complaining only of fatigue. During the subsequent hospitalization and follow-up visits 6 months later, she did not exhibit these symptoms again.

**Table 4:** The acupuncture prescription.

Chinese acupuncture points	International code list of acupoints
Neiguan (内关)	PC6
Shuigou (水沟)	DU26
Sanyinjiao (三阴交)	SP6
Baihui (百会)	DU20
Yintang (印堂)	EX-HN3
Taichong (太冲)	LR3
Hegu (合谷)	LI4
Jiquan (极泉)	HT1

**Table 5:** The prescription for removing phlegm and inducing resuscitation.

TCM	Dose (g)
Pinellia	10
Dried tangerine peel	15
Tuckahoe	15
Codonopsis pilosula	20
Yam	15
Coptis chinensis	5
Fructus amomi	10
Semen coicis	20
Acorus gramineus Soland.	20
Asarum sagittarioides	10
polygala root	15
Lilium brownii	15
Leucaena	10
Artemisia apiacea	15
Licorice	5

TCM = traditional Chinese medicine.

## 3. Discussion

Catatonia is a neuropsychiatric syndrome characterized by psychomotor abnormalities caused by a wide variety of brain-functioning disorders.^[3]^ Karl Kahlbaum, in the 1870s, coined the term “catatonia” for a syndrome characterized by bizarre motor behavior, impaired volition, and vegetative abnormalities.^[4]^ Although abnormalities of psychomotor function are common in schizophrenia, catatonic symptoms are not unique to the disorder. Indeed, it can also be observed in organic encephalopathies, depression, psychotic disorders with hypsarrhythmia, and acute stress responses. Stupor, immobility, mutism, and rigidity that occurred in this patient are part of the typical catatonic symptoms. Catatonia can be as prevalent as 8.9% among patients aged 65 and older in medical departments seeking the advice of a psychiatry liaison service.^[5]^ In addition to careful and repeated observation, the neuropsychiatric examination requires a screening instrument, such as the BFCRS, to identify the catatonic syndrome. Although research on the treatment of catatonia is scarce, it is known that BZDs, such as lorazepam, are the first-line treatment for catatonia. In addition, electroconvulsive therapy has been reported.^[6]^

**Figure 4.**
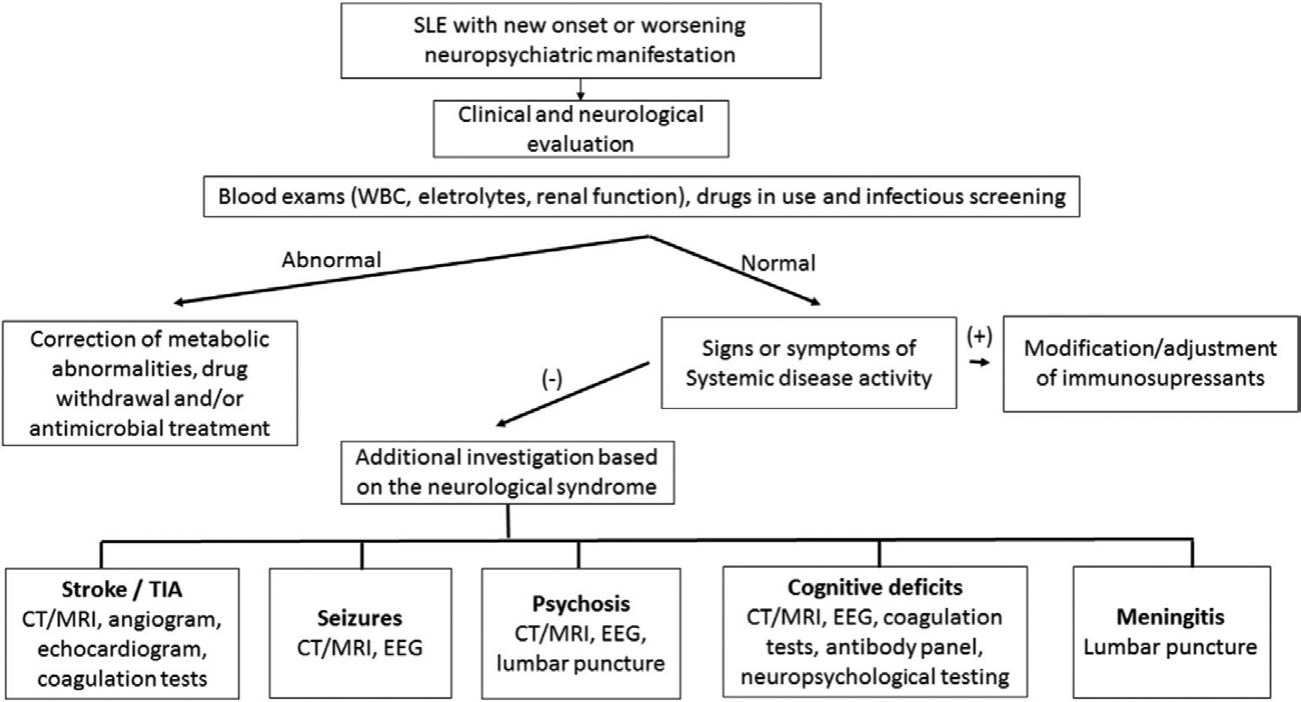
The approach for evaluation of SLE patients presenting with new onset or worsening NP symptoms. (Source of reference: de Amorim JC et al, Mimickers of neuropsychiatric manifestations in systemic lupus erythematosus, Best Practice & Research Clinical Rheumatology, https://doi.org/10.1016/j.berh.2019.01.020). MRI = magnetic resonance imaging, NP = neuropsychiatric, SLE = systemic lupus erythematosus.

NP events are common in patients with SLE, but attributing NP events to SLE necessitates a comprehensive investigation and the exclusion of alternative causes.^[7]^Indeed, only 33% of such occurrences are directly attributed to it.^[8]^ Autoimmune-mediated inflammatory injury and vascular injury are separate pathogenetic mechanisms responsible for NPSLE.^[9]^ Correspondingly, the presence of active disease and autoantibodies is a significant risk factor for NP events. 1999 saw the publication of a consensus statement by the American College of Rheumatology that defined 19 NP syndromes (Table 6). Because of the heterogeneity of clinical manifestations, there is no diagnostic gold standard for NPSLE.^[10]^ Certain clinical, laboratorial, and neuroradiological features are extremely helpful to assist in determining possible etiologies and the differential diagnosis.^[11]^ More recently, different attribution models have been developed to help determine if the NP event is due to SLE, and some researchers have summarized the evaluation strategy for SLE patients presenting with new onset or worsening NP symptoms (Fig. 4). Nonetheless, the primary step of the diagnostic process would still be to investigate thoroughly, categorize the NP manifestations, and exclude other common causes so called “mimickers.” CSF examination (primarily to rule out infection), autoantibody profiling, neuroimaging to evaluate brain structure and function, and neuropsychological evaluation may be performed.^[8]^

**Table 6:** Clinical syndromes in NPSLE.

Syndromes	Central nervous system	Peripheral nervous system
Neurological syndromes	Seizure disorder	Autonomic disorders
	Aseptic meningitis	Mononeuropathy
	Demyelinating syndromes	Myasthenia gravis
	Myelopathy	Polyneuropathy
	Headache	Cranial neuropathy
	Cerebrovascular disease	Guillian Barre syndrome
	Movement disorders	Plexopathy
Neuropsychiatric syndromes	Anxiety disorders	
	Psychosis	
	Acute confusional state	
	Cognitive dysfunction	
	Mood disorders	

NPSLE = neuropsychiatric systemic lupus erythematosus.

As in this case, we endeavored to identify the “mimickers.” She had SLE with the long-term use of hormones and immunosuppressive agents, which increased her risk of infection. In fact, microbiological cultures revealed that she was suffering from a bacterial and fungal infection simultaneously. There were no obvious abnormalities in cranial MRI, electroencephalography, and CSF examination, as well as in blood gas analysis and an electrolyte profile, and we could basically exclude metabolic abnormalities, malignancies, and infections or organic lesions of the nervous system, such as meningitis, stroke, etc. It also needed to be considered whether the adverse effects of medication such as antibiotics were a cause and, in uncertain cases, whether they could be suspended or replaced with other drug alternatives. As a result, we substituted azithromycin for the suspected moxifloxacin. But the negative results for autoantibodies related to NPSLE and the SLEDAI-2000 score continued to confound us. Given the intense mental stimulation, the likelihood of a psychiatric cause was relatively high. We shifted our focus to the symptoms and performed the BFCRS score.

As stated previously, we refrained from administering the medication out of concern for impairment of renal function and the possibility of further deterioration of the coma. It should be noted that supportive care, TCM, and acupuncture therapy were utilized in the following 2 days after a carefully and meticulously comprehensive assessment of the condition.

In conclusion, identification of the most common mimic disorders and accurate diagnosis of NPSLE are essential for guiding appropriate treatment and preventing long-term complications or mortality in SLE patients. Although this error is primarily based on excluding diagnosis and evaluation, it is encouraging to see that researchers are optimizing the diagnostic process in continuous clinical summaries in an effort to reduce the rate of misdiagnosis. This case also provides a reference for TCM and acupuncture treatment of a catatonic disorder. When clinical treatment options are limited, a variety of combination strategies, such as TCM and acupuncture, can play an important role.

**Table 1:** Results of blood tests during hospitalization and at catatonia symptoms.

Variable	At admission	At catatonia symptoms	Reference range
Leukocyte count	0.39 × 10^9/L	1.13 × 10^9/L	3.5–9.5 × 10^9/L
Neutrophil count	0.21 × 10^9/L	0.80 × 10^9/L	1.8–6.3 × 10^9/L
Erythrocyte count	2.39 × 10^12/L	2.28 × 10^12/L	3.8–5.1 × 10^12/L
Platelet count	149 × 10^9/L	122 × 10^9/L	100–300 × 10^9/L
Hemoglobin	75 g/L	71 g/L	115–150 g/L
C-reactive protein	201.81 mg/L	58.36 mg/L	0–5 mg/L
K	3.64 mmol/L	3.70 mmol/L	3.5–5.5 mmol/L
Na	128.6 mmol/L	148.2 mmol/L	137–147 mmol/L
Cl	91.0 mmol/L	113.7 mmol/L	99–110 mmol/L
Ca	2.23 mmol/L	2.40 mmol/L	2.11–2.52 mmol/L
Alanine aminotransferase	8 U/L	15 U/L	7–40 U/L
Aspartate aminotransferase	19 U/L	12 U/L	13–35 U/L
Alkaline phosphatase	54 U/L	53 U/L	50–135 U/L
Serum total protein	56.7 g/L	55.1 g/L	65–85 g/L
Albumin	34.2 g/L	34.5 g/L	40–55 g/L
Creatinine	99.0 μmol/L	109.8 μmol/L	41–81 μmol/L
Urea nitrogen	12.94 mmol/L	17.63 mmol/L	3.1–8.8 mmol/L
Uric acid	224 μmol/L	344 μmol/L	155–357 μmol/L
Glucose	4.80 mmol/L	5.23 mmol/L	3.89–6.11mmol/L
Procalcitonin	0.44 ng/mL	0.14 ng/mL	0–0.05 ng/mL

**Table 2:** Results of CSF examination.

Variable	Results	Units	Reference range
Appearance			
Color	Colorless		
Transparency	Clear		
Clot	(-)		
Rivalta test			
Protein qualitative	(-)		(-)
Microscope examination			
Leukocyte count	0.2	10^6/L	
Erythrocyte count	0.2	10^6/L	
Biochemical examination			
Alanine aminotransferase	<7	U/L	0–15
Aspartate aminotransferase	20	U/L	0–20
Alkaline phosphatase	37	U/L	0–40
Phosphocreatine kinase	1	U/L	0.5–2
Adenylate deaminase	<0	U/L	0–8
Lactic acid	1.5	mmol/L	0.6–2.2
Protein	201	mg/L	200–400
Cl	127.4	mmol/L	120–130
Glucose	3.25	mmol/L	2.5–4.4

**Table 3:** Results of blood gas analysis.

Variable	Results	Units	Reference range
Temperature	37.0	°C	
pH	7.43		7.35–7.45
Partial pressure of carbon dioxide	34.0	mm Hg	35–45
Partial pressure of oxygen	136.0	mm Hg	80–100
Glucose	5.4	mmol/L	3.3–5.3
Ca	1.30	mmol/L	1.15–1.35
Lactic acid	0.6	mmol/L	0.5–2.2
Hematocrit	33.0	%	37–49
Measured bicarbonate	22.6	mmol/L	21.4–27.3
Standard bicarbonate	24.0	mmol/L	21.3–24.8
Total carbon dioxide	23.6	mmol/L	24–32
Intracellular surplus base	20	mmol/L	3–3
Extracellular surplus base	37	mmol/L	3–3
Oxygen saturation	99.0		91.9–99
Total hemoglobin	102.0	g/L	110–160

## Acknowledgments

The authors are grateful to the patient for allowing publication of her case details.

## Author contributions

**Investigation:** Lizeyu Lv, Yong Lin, Liangbin Zhao.

Project administration: Lizeyu Lv, Wen Xiao.

Supervision: Liangbin Zhao.

Visualization: Yu Zhang.

Writing – original draft: Lizeyu Lv.

Writing – review & editing: Mingquan Li, Liangbin Zhao.
